# Supplementary material for: Resolving the full spectrum of human genome variation using Linked-Reads
Source: Genome Res. 2019 Apr;29(4):635–45. doi: 10.1101/gr.234443.118 (PMC6442396; doi:10.1101/gr.234443.118)
Supplement: Supplemental Material [file supp_gr.234443.118_Supplemental_longranger-2.2.zip › longranger-2.2.tar.bz2!/lr2.2-public/lib/stan/cmdstan/stan/doc/api/html/whitespace__grammar__def_8hpp_source.html]

Stan: src/stan/gm/grammars/whitespace\_grammar\_def.hpp Source File


|  |  |
| --- | --- |
|  | Stan  2.5.0  probability, sampling & optimization |


- Main Page
- Related Pages
- Namespaces
- Classes
- Files

- File List
- File Members

All Classes Namespaces Files Functions Variables Typedefs Enumerations Enumerator Friends Macros Pages

- src
- stan
- gm
- grammars

whitespace\_grammar\_def.hpp

Go to the documentation of this file.

1 #ifndef STAN\_\_GM\_\_PARSER\_\_WHITESPACE\_GRAMMAR\_DEF\_\_HPP

2 #define STAN\_\_GM\_\_PARSER\_\_WHITESPACE\_GRAMMAR\_DEF\_\_HPP

3

4 #include <boost/spirit/include/qi.hpp>

5

6 #include <stan/gm/grammars/whitespace\_grammar.hpp>

7

8 namespace stan {

9

10  namespace gm {

11

12  template <typename Iterator>

13  whitespace\_grammar<Iterator>::whitespace\_grammar()

14  : whitespace\_grammar::base\_type(whitespace)

15  {

16  using boost::spirit::qi::omit;

17  using boost::spirit::qi::char\_;

18  using boost::spirit::qi::eol;

19  whitespace

20  = ( ( omit["/\*"]

21  >> \*(char\_ - "\*/") )

22  > omit["\*/"] )

23  | ( omit["//"] >> \*(char\_ - eol) )

24  | ( omit["#"] >> \*(char\_ - eol) )

25  | boost::spirit::ascii::space\_type()

26  ;

27  }

28

29  }

30

31 }

32

33

34

35 #endif

stan::gm::whitespace\_grammar::whitespace

boost::spirit::qi::rule< Iterator > whitespace

**Definition:** whitespace\_grammar.hpp:16

stan::gm::whitespace\_grammar

**Definition:** whitespace\_grammar.hpp:11

whitespace\_grammar.hpp

stan::gm::whitespace\_grammar::whitespace\_grammar

whitespace\_grammar()

**Definition:** whitespace\_grammar\_def.hpp:13


---

[ Stan Home Page ]

*© 2011–2014,
Stan Development Team.*
